# Vitamin D Supplementation for Patients with Chronic Kidney Disease: A Systematic Review and Meta-analyses of Trials Investigating the Response to Supplementation and an Overview of Guidelines

**DOI:** 10.1007/s00223-021-00844-1

**Published:** 2021-04-25

**Authors:** Marilena Christodoulou, Terence J. Aspray, Inez Schoenmakers

**Affiliations:** 1 Medical School, University of East Anglia, Norwich, UK; 2 University of Newcastle Upon Tyne, Freeman Hospital, Bone Clinic, Newcastle, UK; 3 Department of Medicine, Norwich Medical School, Faculty of Medicine and Health Sciences, University of East Anglia, Norwich Research Park, Norwich, NR4 7TJ UK

**Keywords:** Vitamin D supplementation, Vitamin D deficiency, Chronic kidney disease, Systematic review, Guidelines

## Abstract

A large proportion of patients with chronic kidney disease (CKD) are vitamin D deficient (plasma 25-hydroxyvitamin D (25(OH)D) < 25 or 30 nmol/L per UK and US population guidelines) and this contributes to the development of CKD–mineral bone disease (CKD–MBD). Gaps in the evidence-base for the management of vitamin D status in relation to CKD–MBD are hindering the formulation of comprehensive guidelines. We conducted a systemic review of 22 RCTs with different forms of vitamin D or analogues with CKD–MBD related outcomes and meta-analyses for parathyroid hormone (PTH). We provide a comprehensive overview of current guidelines for the management of vitamin D status for pre-dialysis CKD patients. Vitamin D supplementation had an inconsistent effect on PTH concentrations and meta-analysis showed non- significant reduction (*P* = 0.08) whereas calcifediol, calcitriol and paricalcitol consistently reduced PTH. An increase in Fibroblast Growth Factor 23 (FGF23) with analogue administration was found in all 3 studies reporting FGF23, but was unaltered in 4 studies with vitamin D or calcifediol. Few RCTS reported markers of bone metabolism and variations in the range of markers prevented direct comparisons. Guidelines for CKD stages G1–G3a follow general population recommendations. For the correction of deficiency general or CKD-specific patient guidelines provide recommendations. Calcitriol or analogues administration is restricted to stages G3b–G5 and depends on patient characteristics. In conclusion, the effect of vitamin D supplementation in CKD patients was inconsistent between studies. Calcifediol and analogues consistently suppressed PTH, but the increase in FGF23 with calcitriol analogues warrants caution.

## Introduction

Chronic kidney disease (CKD) is an international public health problem affecting 5–10% of the world population [1]. It is defined on the basis of an estimated glomerular filtration rate (eGFR) and values less than 60 mL/min/1.73 m^2^ are considered the threshold for CKD [2–4]. Many are undiagnosed and asymptomatic. Only in the UK there are approximately 1 million undiagnosed CKD patients [5]. CKD results in changes in vitamin D metabolism, calcium and phosphate homeostasis and bone metabolism [6, 7]. This leads to CKD metabolic bone disease (CKD–MBD) [7].

The prevalence of vitamin D deficiency is higher in CKD patients than in the general population [6]. Vitamin D status is most commonly assessed on the basis of the plasma concentration of 25-hydroxyvitamin D (25(OH)D or calcidiol). Vitamin D deficiency thresholds for population health are defined as 25(OH)D serum levels < 25 or 30 nmol/L per UK and US guidelines [8, 9]. Thresholds for patient management are mostly set higher.

Many factors contribute to the high prevalence of vitamin D deficiency in CKD patients. Dependent on the stage of CKD and presence or severity of secondary hyperparathyroidism (SHPT), supplementation with vitamin D, analogues or its active form is recommended. Guidelines for management of CKD patients combine those for the general population, general patient management and guidelines specific for CKD patients. This requires detailed knowledge of recommendations on vitamin D for the protection of population health, patient care and the specific guidelines to correct vitamin D deficiency. Due to differences in recommendations between advisory bodies, this is perceived to be confusing.

There are still many gaps in the evidence base for the management of vitamin D status in relation to CKD–MBD, including the role of vitamin D in the aetiology and management of SHPT, altered bone metabolism, bone density and integrity and fracture risk. These include the altered dose–response to vitamin D supplementation and the response in parathyroid hormone (PTH) in these patients. The optimal concentration ranges of PTH and 25(OH)D for the management and prevention of CKD–MBD are also not well established for each stage of CKD.

Here we briefly summarise alterations in vitamin D metabolism and bone metabolism with CKD and gaps in the evidence base. We present the findings of a systematic review of recent RCTs with different forms of vitamin D in CKD patients with a focus on CKD–MBD related outcomes. Meta-analyses were conducted to provide estimates of the effectiveness of supplementation on plasma PTH concentrations. Secondly, we provide a comprehensive review of the available guidelines for adult pre-dialysis renal patients for the management of vitamin D status and SHPT. We generated a road map, tabulating guidelines from different organizations on the form and dosages of vitamin D recommended to prevent or correct vitamin D deficiency and target values of plasma 25(OH)D concentrations, according to the different stages of CKD. Population guidelines are summarized as part of this road map where they apply to CKD patients.

### The Physiology of Altered Vitamin D and Bone Metabolism with CKD

The alterations in vitamin D metabolism [6], calcium and phosphate homeostasis and bone metabolism [6] with CKD is multifactorial and associated with CKD–MBD [7].

A high proportion, 70–80%, of CKD patients have a plasma 25(OH)D concentration below 50 nmol/L [6] and the majority well below the concentration recommended for patients with renal impairment (> 75 nmol/L), if not treated. Many factors contribute to the high prevalence of vitamin D deficiency in CKD patients and changes in vitamin D metabolism occur at several levels (Fig. 3).

Supply is decreased as a result of lower cutaneous vitamin D production due to skin hyperpigmentation, ageing, sun avoidance and dietary restrictions [10]. Losses are increased with proteinuria, when vitamin D binding protein and albumin and vitamin D metabolites bound to these proteins are lost in urine [10]. Hepatic conversion of vitamin D into 25(OH)D is reported to be suppressed in CKD patients [11–13]. Accordingly, the dose–response appears to be lower than in healthy individuals, although this is poorly characterized.

With the loss of functional renal tissue the capacity to convert 25(OH)D to 1,25(OH)_2_D (1,25 dihydroxy vitamin D or calcitriol) is reduced leading to a decline in plasma 1,25(OH)_2_D [11]. Also the renal capacity to internalise 25(OH)D may be impacted, reducing its availability of 25(OH)D. In healthy people, plasma 25(OH)D concentrations between 15 and 40 nmol/L are thought to be required to ensure there is no substrate limitation for renal 1,25(OH)_2_D production [14]. In people with CKD, higher concentrations may be required.

Plasma PTH increases in response to impaired 1,25(OH)_2_D production and calcium malabsorption in combination with increased resistance of the kidneys and bone to PTH, due to a downregulation of PTH receptor type 1 (PTHR1) [15]. An increase in plasma phosphate and Fibroblast Growth Factor-23 (FGF23) further stimulate PTH secretion.

The plasma concentration of FGF23 increases in early stages of CKD, before an increase in plasma phosphate is detectable [16]. FGF23 is a phosphaturic hormone which is predominantly produced by osteocytes and acts in the kidneys to increase phosphate excretion [16]. It requires the co-factor αKLOTHO, the expression of which decreases with ageing and renal impairment, thereby decreasing FGF23 receptor activation. FGF23 also has other functions. FGF23 stimulates the catabolism of both 25(OH)D and 1,25(OH)_2_D. FGF23 also downregulates the expression of 1a(OH)ase, suppressing the production of renal 1,25(OH)_2_D. Further, FGF23 can stimulate PTH secretion [16], although the mechanism of this FGF23-PTH interaction is not well understood. An increased plasma FGF23 concentration is associated with soft tissue calcification, increased risks of cardiovascular disease (CVD) and the promotion of CKD–MBD [16].

CKD–MBD has a heterogeneous phenotype due to the involvement of several underlying mechanisms, in which SHPT plays an important role [35]. CKD–MBD can either be characterised by an increase or a decrease in bone turnover, but may also be normal. An increase can lead to osteomalacia, which is characterized by the excessive presence of undermineralised bone tissue and osteoporosis defined as a low bone mineral density (*Z*-score − 2.5 SD) and loss of bone integrity. A decrease in bone remodelling leads to adynamic bone disease increasing fracture risk [1]. CKD–MBD is therefore generally characterized by a decrease of bone integrity, increased fracture risk and calcification of soft tissues [7] and alterations in bone turnover markers [1]. In the management of SHPT and CKD–MBD, maintenance of a sufficient vitamin D status is recognized as an important target.

### Clinical Trials of Vitamin D in CKD Patients and Gaps in the Evidence-Base

There are gaps in the evidence base for the management of vitamin D status in relation to CKD–MBD, i.e. SHPT, altered bone metabolism, bone density and integrity and fracture risk. These include the dose- response to vitamin D supplementation and the response in PTH. The optimal concentration ranges of PTH and 25(OH)D for the management and prevention of CKD–MBD are not well established for each stage of CKD. This is reflected in the guidelines for the management of vitamin D status in CKD–MBD.

The limited number of randomized controlled trials reporting the effects of treatment with vitamin D or its analogues on CKD–MBD related outcomes provided conflicting results [18–31]. Several studies also reported the effect of supplementation on renal function and proteinuria [32] and markers of endothelial and cardiovascular function and inflammation [33, 34]. The effects were shown to depend on the stage of the disease [35, 36]. Adverse treatment effects were reported, particularly with active vitamin D and analogues and includes hypercalcemia [37, 38], adynamic bone disease and increased FGF23 levels [39].

## Systematic Review and Meta-analyses of Randomised Controlled Trials

In this systematic review we aimed to summarize the findings of the most recent randomized controlled trials reporting the effects of vitamin D or its analogues, conducted with pre-dialysis CKD patients and that report 25(OH)D, PTH, markers of calcium and phosphate and/or bone metabolism. Where provided, we also summarized adverse effects and other outcomes that may be relevant for vitamin D metabolism (e.g. proteinuria) or the effects of interventions on markers of vascular health. Findings are grouped according to form of vitamin D given, preceded by a short description of their characteristics. Meta-analyses were conducted to provide estimates of the effectiveness of supplementation on plasma PTH concentrations. There were insufficient studies and data to conduct meaningful meta-analyses for markers of bone turnover or FGF23.

## Methods

### Search Strategy

We searched for published studies indexed in MEDLINE, EBSCO, Science direct and PubMed from inception of 2003, the year that NKF KDOQI guidelines were published, to October of 2020. Search terms used: vitamin D, oral vitamin D, vitamin D supplementation, vitamin D analogues, paricalcitol, calcifediol, calciferol, ergocalciferol, chronic kidney/renal disease, renal/kidney impairment, impaired kidney/renal failure, RCT, randomised controlled trials. Search terms for outcomes were not used in order not to limit search results. Instead, papers were selected on basis of relevant outcomes. English was applied as a language limitation. Only full text published manuscripts were included. References were hand searched for additional publications.

### Inclusion Criteria

**Fig. 1:**
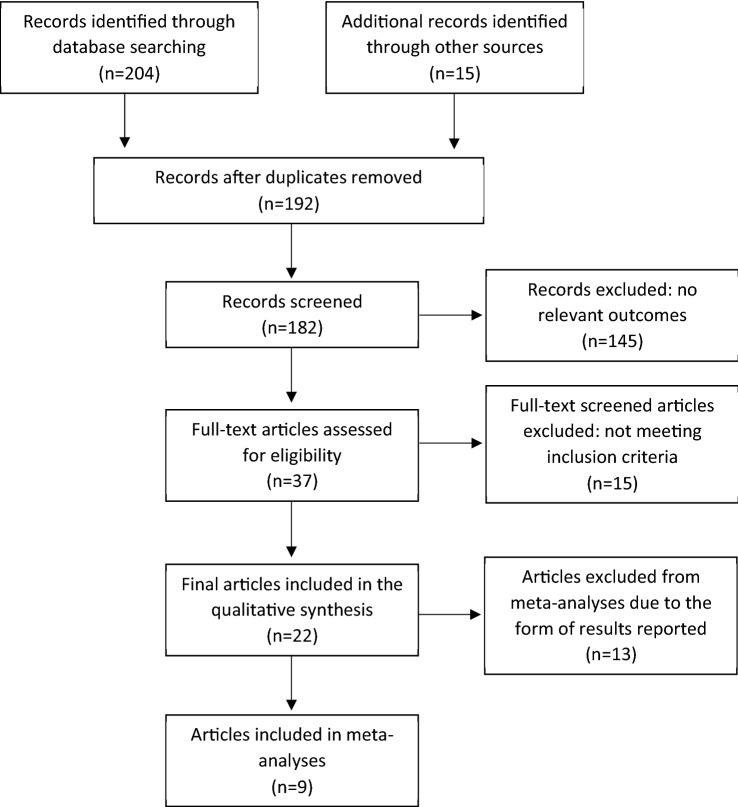
Flow chart of systematic search and literature selection. Adjusted from PRISMA protocol 2019

The detailed process of study selection presented on Fig. 1. Only randomized controlled trials (RCT) were included that used any type of vitamin D in non-dialysis CKD patients (> 18 years old) and studies at any stage of the disease were considered. Studies were included if placebo controlled, compared 2 or more treatments or were randomized cross-over studies. Studies had to include a definition of dosage and duration of the vitamin D administered and outcomes related to CKD–MBD. This systematic search provided 22 RCTs (Table 1).

### Quality Assessment

The 22 studies were assessed for their methodological quality using the Van Tulder et al. criteria list [40] by two independent investigators (Table 1). High quality studies were defined with score ≥ 8 and low quality with score ≤ 7.18. Scores are provided in Table 1.

**Table 1:**
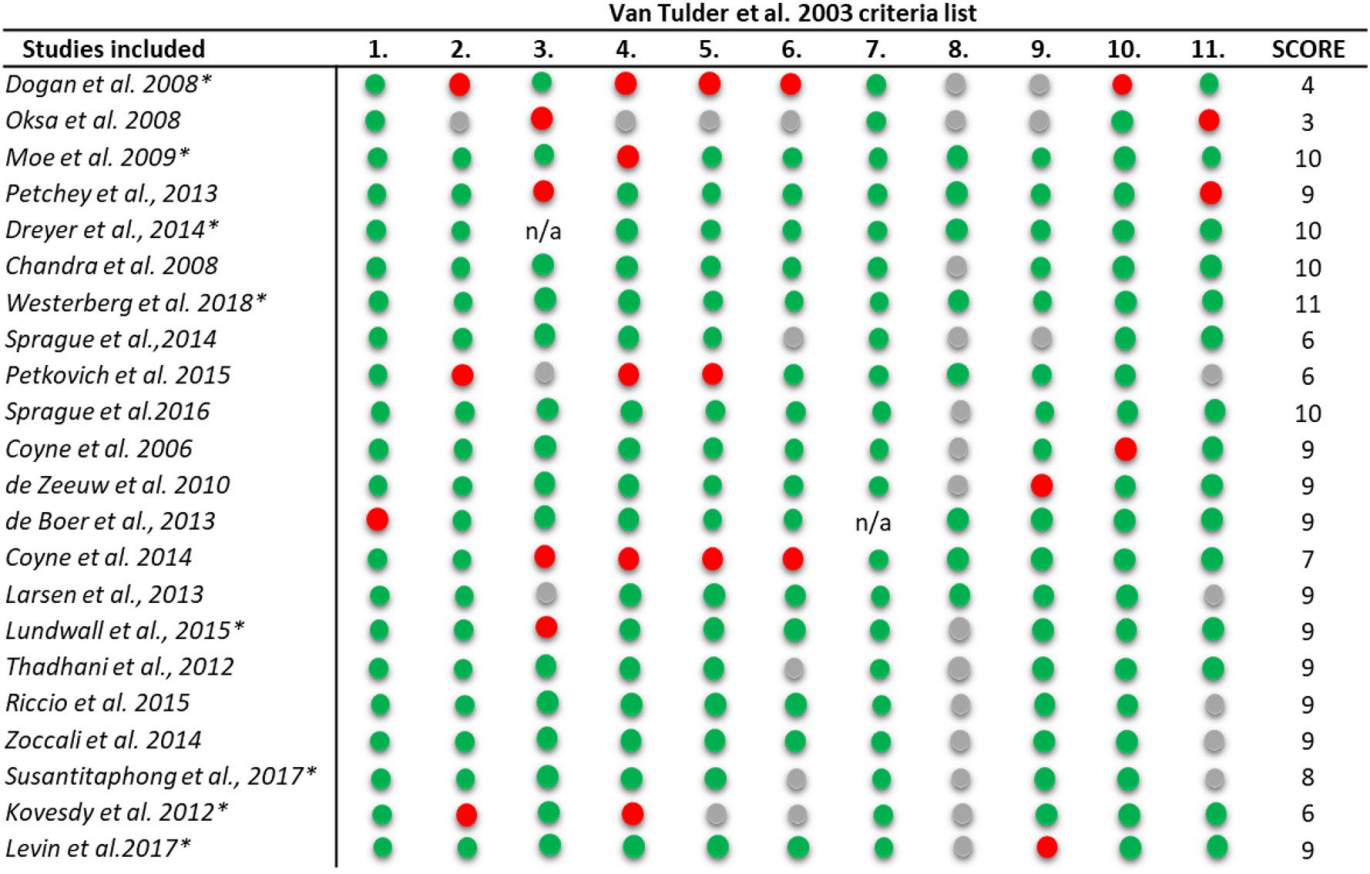
Quality assessment of the RCTs included in the systematic review according to Tulder et al. ^[40]^

Green: Yes, Red: No, Grey: Unknown. 1. Was the method of randomization adequate? 2. Was the treatment allocation concealed? 3. Were the groups similar at baseline regarding the most important prognostic indicators? 4. Was the patient blinded to the intervention? 5. Was the care provider blinded to the intervention? 6. Was the outcome assessor blinded to the intervention? 7. Were co-interventions avoided or similar? 8. Was the compliance acceptable in all groups? 9. Was the drop−out rate described and acceptable? 10. Was the timing of the outcome assessment in all groups similar? 11. Did the analysis include an intention−to−treat analysis?

*Studies included in the meta−analyses

### Meta-analyses

Plasma PTH concentrations were converted into uniform units (pg/ml). Random effect models were used for all meta-analyses because of differences in form, dosages, duration and study population. Heterogeneity was assessed with the *I*^2^ test. Separate analyses were conducted for studies using the precursor of 1,25(OH)_2_D (i.e. vitamin D or 25(OH)D) and those with 1,25(OH)_2_D or analogues. The decision to separate these studies was based on high heterogeneity (Chi^2^ *P* = 0.001: *I*^2^ = 65%) resulting when both forms were analyzed together and on biological plausibility. Analyses used the reported mean and standard deviations or confidence intervals, either between treatment groups and/or placebo as appropriate. Not all studies reported sufficient detail for inclusion. Some studies could not be included because of the type of data reported (e.g. as % difference between groups or geometric mean or median values for non-normally distributed data). In view of the limited number of placebo–controlled studies, baseline data were used as comparator for some studies. Four studies reported baseline and post-placebo treatment data and none of these individual studies reported a significant change in PTH and differences were small. This was confirmed by meta-analysis showing a non-significant change (results not shown).

Meta-analyses were performed using RevMan (version 5.4; non-Cochrane Collaboration). A *P*-value of ≤ 0.05 was considered significant.

## Results

### RCTs with Vitamin D Supplementation

Vitamin D is the inactive precursor of 1,25(OH)_2_D and exists in two forms, vitamin D_3_ (cholecalciferol) and D_2_ (ergocalciferol). Vitamin D is the most commonly used form for the prevention and treatment of deficiency in both the general population and patient groups. Vitamin D_3_ supplementation leads to a somewhat longer sustained increase of 25(OH)D, but otherwise the metabolism of D_3_ and D_2_ is identical.

**Fig. 2:**
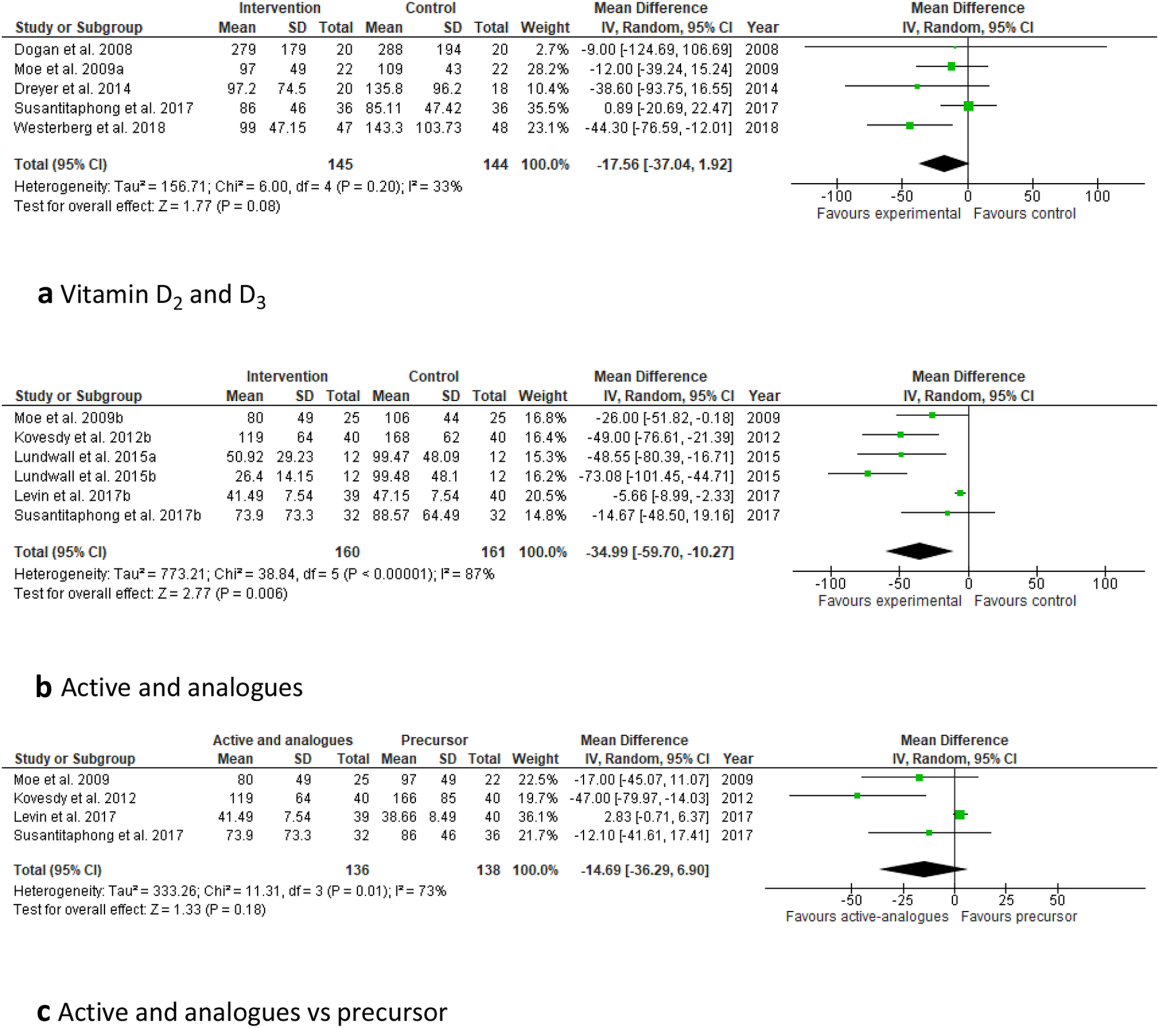
Forest plots of the effect of different forms of vitamin D supplementation on PTH concentrations in CKD patients. Top: **a** effect of vitamin D_2_ or D_3_ on PTH. Middle: **b** effect of calcitriol or analogues on PTH. Bottom: **c** effect of calcitriol or analogues (active) versus Vitamin D_2_ or D_3_ (precursors) on PTH. PTH concentrations are given in pg/mL. Details on form and dosages are given in Table 1

Nine of the RCTs included in this systematic review used vitamin D_3_ (six studies) [25, 41–45] or D_2_ (three studies) [21, 29, 46] supplementation (Table 2). All studies has a small sample size (*n* < 100). In some of these studies the group that received Vitamin D served as the reference group. They were all conducted with CKD patients stages 3–4. The doses varied from 2000 to 4000 IU daily or 40,000–50,000 IU weekly and the duration was between 1 and 12 months. All nine studies found a significant increase in plasma 25(OH)D concentrations. Four [41–43, 46] of these studies observed a significant reduction of PTH, whilst in the remaining five, no significant change of PTH in response to the vitamin D supplementation was found [25, 29, 44, 45]. The small sample sizes, differences in dosages and duration of supplementation may explain these inconsistent results; higher doses show to be more effective in suppressing PTH. Only five of these studies provided sufficient detail for inclusion in meta-analysis, which showed a non-statistically significant decrease in PTH of 18 pg/mL (CI − 37 to 2; *P* = 0.8 see Fig. 2a). When also two studies with calcifediol were considered, the effect on PTH was highly significant (*P* < 0.0001), but heterogeneity was substantial (*I*^2^ = 60%) (not shown).

**Table 2:** Study characteristics and outcomes included in the systematic review

Authors	Country	Study population	Intervention	Outcomes
Vitamin D*				
Dogan et al. [41]	Slovakia	40 predialysis CKD patients (stage 3 and 4), PTH < 200 pg/mL. No use of phosphate binders	300,000 IU D_3_/month for 1 month or placebo Route: oral	Treatment group: ↑ 25(OH)D, ↓ iPTH, ↔ serum calcium, ↔ serum phosphorus, ↔ ALP
Oksa et al. [42]	UK	87 CKD patients (stage 2–4) (including hypertensive, diabetic and vitamin D insufficient/ deficient CKD patients). Use of phosphate binders (calcium-based). No calcimimetics use	5000 or 20,000 IU/week D_3_ for 12 months Route: oral	↑ 25(OH)D, ↓ iPTH in both treatment groups, ↔ serum calcium, ↔ serum phosphate, ↔ urinary calcium, ↔ urinary phosphate
Petchey et al. [25]	USA	28 CKD patients (stage 3–4), 25(OH)D < 150 nmol/L	2000 IU/day D_3_ or placebo for 6 months Route: oral	↑ 25(OH)D, ↑ 1,25(OH)_2_D, ↔ PTH, ↔ serum calcium, ↔ serum phosphate, ↔ insulin sensitivity
Dreyer et al., [29]	USA	38 CKD patients (stage3-4), non-diabetic, 25(OH)D < 40 nmol/L	50,000 IU/week D_2_ for 1 month followed by 50,000 IU/month D_2_ for 5 months or placebo for 6 months Route: oral	↑25(OH)D, ↔ PTH, ↔ calcium, ↔ phosphate, ↔ blood pressure, ↔ left ventricular mass index. Improvement in endothelium dependent microcirculatory vasodilation
Chandra et al. [44]	Turkey	34 CKD patients (stage 3 and 4), 25(OH)D < 75 nmol/L and SPTH (PTH > 70 pg/mL). No calcimimetic use	50,000 IU/week D_3_ or placebo for 12 weeks Route: oral	↑ 25(OH)D, ↔ 1,25(OH)_2_D, ↔ PTH, ↔ serum calcium, ↔ BAP, ↔ TRAP5b, ↔ CTX
Westerberg et al. [45]	Sweden	95 CKD patients (stage 3–4), 25(OH)D < 75 nmol/L, PTH > 64.1 pg/mL. No calcimimetic use	8000 IU/day D_3_ or placebo for 12 weeks Route: oral	↑25(OH)D, ↑1,25(OH)_2_D, ↔ PTH but it was significantly lower than the mean value of the placebo group. ↑ calcium, ↔ phosphate and ↔ FGF23
*25 (OH) Vitamin D—calcifediol*				
Sprague et al. [22]	USA	78 CKD patients (stage 2–4), 25(OH)D < 75 nmol/L, iPTH > 70 pg/mL	ER calcifediol (30, 60 or 90 µg/day) or placebo for 6 weeks Route: oral	Dose dependent ↑ 25(OH)D and ↓ iPTH, serum ↔ calcium, ↔ serum phosphorus ↔ FGF23
Petkovich et al. [48]	USA	29 CKD patients (stage 3–4), 25(OH)D < 75 nmol/L, SHPT	Single oral dose of ER calcifediol (450 mg or 900 mg) or a single bolus IV injection of calcifediol (448 mg) and monitoring for 42 days Route: oral and iv	*ER calcifediol (450 mg or 900 mg):* ↔ 25(OH)D and ↔ 1,25(OH)_2_D, ↔ PTH compared to IV group *ER calcifediol (900 mg):* ↔ 25(OH)D and ↔ 1,25(OH)_2_D, ↔ 24,25(OH)_2_D, ↓iPTH after 72 h compared to IV group *IV injection group*: ↑25(OH)D, ↑1,25(OH)_2_D, ↑24,25(OH)_2_D, ↔ iPTH ↔ calcium in all treatment groups
Sprague et al. [49]	USA	429 CKD patients (stage 3–4), 25(OH)D 25–75 nmol/L, SHPT (≥ 85 and < 500 pg/mL)	ER calcifediol (30 or 60 μg/day) or placebo for 26 weeks Route: oral	↑25(OH)D, ↓PTH in both treatment groups compared to placebo, ↔ serum calcium, ↔ serum phosphorus, ↔ FGF23
*1,25(OH)*_*2*_ vitamin D or Vitamin D analogues**				
Coyne et al. [57]	USA	220 CKD patients (stages 3 and 4) with PTH (> 70 pg/mL)	Paricalcitol capsules (Dosing was based on serum iPTH, calcium, and phosphorus levels) 3/week or 1/day or placebo for 24 weeks Route: oral	↓ iPTH compared to placebo, ↔ urinary calcium,, ↔ urinary phosphorus, ↓ BAP, ↓ osteocalcin, ↓ urinary pyridinoline compared to baseline
de Zeeuw et al. [53]	USA	281 patients with type 2 diabetes, nephropathy (stages 1–4) and PTH 35–500 pg/mL	1 μg paricalcitol/day, 2 μg paricalcitol/day or placebo for 24 weeks Route: oral	↑25(OH)D, ↓iPTH in both treatment groups
de Boer et al. [26]	USA, Poland	22 non-diabetic CKD patients (stage 3–4). No phosphate binders use	Cross-overs study with paricalcitol or placebo for 8 weeks (washout 8 weeks between arms) Route: oral	↓ 25(OH)D, ↓ 1,25(OH)2D, ↑ 24,25(OH)D, ↓ PTH, ↑ serum calcium, ↔ serum phosphorus, ↑ FGF23, ↔ insulin sensitivity
Coyne et al. [58]	Germany, Greece, Italy, Netherlands, Poland, Portugal, Spain, USA, Taiwan	110 CKD patients (stage 3–4), PTH > 120 pg/mL. Use of phosphate binders	0.25 μg/d 1,25(OH)_2_D or 1 μg/day of paricalcitol for 24 weeks Route: oral	↓ iPTH, ↔ serum calcium, ↔ serum phosphorus, ↓ALP compared to baseline
Larsen et al. [28]	Sweden	26 CKD patients (stage 3–4), non-diabetic, albuminuria (urine albumin > 30 mg/L)	paricalcitol (2 µg/day) or placebo for 6 weeks (crossover design) with a 2 week washout period Route: oral	↔ 25(OH)D, ↓ iPTH, ↑ plasma calcium, ↑ plasma phosphate, ↑ urinary calcium (24 h), ↑ FGF23, ↓ ALP, ↓ albumin excretion rate, ↓ creatinine clearance, ↔ renin, ↔ angiotensin II, ↔ aldosterone
Lundwall et al. [19]	Denmark	36 non-diabetic CKD patients (stage 3–4), PTH 35–500 pg/mL	Paricalcitol (1 µg or 2 µg/day) or placebo for 3 months Route: oral	↔ 25(OH)D, ↓ PTH, ↔ calcium, ↔ phosphate in both treatment groups ↔ albuminuria, ↔ pulse wave velocity, ↔ muscle sympathetic nerve activity, ↓ endothelial function at the placebo and 1 µg treatment group, ↑ blood velocity in both treatment groups
Thadhani et al. [30]	USA	227 CKD patients (stage 3–4), iPTH 50–300 pg/mL and mild- moderate left ventricular hypertrophy	Paricalcitol (2 µg/day) or placebo for 48 weeks Route: oral	↓ PTH,↑ calcium, ↔ phosphate, ↔ left ventricular mass index
Riccio et al. [56]	Italy	60 CKD patients (stage 3b-5), PTH 20–300 pg/mL and anaemia (Hb levels: 10–12.5 g/dL), including use of calcium supplements and phosphate binders	Paricalcitol (1 μg/ day) or 1,25(OH)_2_D (0.5 μg/ every other day) for 6 months Route: oral	↔ PTH, ↔ calcium, ↔ phosphate compared to baseline, ↓GFR in the paricalcitol group. The paricalcitol group had a significant ↑Hb where in 1,25(OH)_2_D group was significantly decrease
Zoccali et al. [55]	Italy	88 CKD patients (stage 3 to 4), PTH > 65 pg/mL. Use of phosphate binders	Paricalcitol (2 μg/ day) or placebo for 12 weeks Route: oral	↑25(OH)D, ↓1,25(OH)_2_D, ↓ PTH, ↑ serum calcium, ↑ serum phosphate,↑ FGF23, ↓ GFR
Kovesdy et al. [46]	USA	80 CKD patients (stage 3–4), 25(OH)D < 75 nmol/L and SHPT. Use of phosphate binders	50,000 IU/week D_2_ titrated to achieve serum 25(OH)D 75 nmol/L or paricalcitol (1 µg/day) for 16 weeks Route: oral	↑25(OH)D in both groups compared to baseline, ↓PTH in paricalcitol group, ↔ serum calcium, ↔ serum phosphorus
Moe et al. [43]	Australia	47 CKD stages 3 and 4 with 25(OH)D < 50 nmol/L and SPTH (> 100 to 150 pg/mL for stage 3 and > 150 to < 400 pg/mL for stage 4). No calcimimetic use	4000 IU/day D_3_ for 1 month, then 2000 IU/day D_3_ for 2 months or doxercalciferol (1 μg/day) for 3 months Route: oral	↑ 25(OH)D in both treatment groups, ↓ PTH in doxercalciferol group, ↔ serum calcium, ↔ serum phosphorus, ↔ urinary calcium
Levin et al. [50]	USA	87 CKD patients (stage 3b-4). Use of phosphate binders (calcium-based)	Calcifediol (5000 IU) or 1,25(OH)_2_D (0.5 μg) or placebo, thrice weekly for 6 months Route: oral	↑25(OH)D in the calcifediol group ↔ 1,25(OH)2D, ↓ PTH, ↔ serum calcium, ↔ serum phosphate, ↔ FGF23 in calcifediol and 1,25(OH)_2_D group
*Combination treatment*				
Susantitaphong et al. [21]	Thailand	68 CKD patients (stage 3–4), 25(OH)D < 75 nmol/L with proteinuria	40,000 IU/week D_2_ plus placebo or 40,000 IU/week D_2_ plus 1,25(OH)_2_D (5 µg two times/ week) for 12 weeks Route: oral	↑ 25(OH)D in both treatment groups (higher in the combined group), ↔ iPTH in D_2_ group, ↓ iPTH levels in the combined treatment group, ↔ serum calcium ↔ serum phosphate. ↓ urine protein-creatinine ratio in both treatment groups

*3 further studies with vitamin D [43, 46, 21] and **further study with calcifediol [50] are listed below

*CKD* Chronic kidney disease, *D3* vitamin D3 or Cholecalciferol, *D2* vitamin D2 or Ergocalciferol, *25(OH)D* 25 hydroxy vitamin D or Calcidiol, *1,25(OH)2D* 1,25 dihydroxy vitamin D or Calcitriol, *24,25(OH)2D* 24,25 dihydroxy vitamin D, *ER* extended release, *PTH* parathyroid hormone, *SHPT* secondary hyperparathyroidism, *FGF23* Fibroblast growth hormone−23, *ALP* alkaline phosphatase, *BAP* bone−alkaline phosphatase, *CTX* C−terminal telopeptide, *TRAP5b* tartrate−resistant acid phosphatase 5b, *GFR* glomerular filtration rate, *Hb* haemoglobin

Conversion factor plasma concentration 25(OH)D in nmol/L to ng/mL: divide by 2.5

Conversion factor Vitamin D in IU to µg: divide by 40

Only three of the studies [25, 44, 45] measured changes in 1,25(OH)_2_D, with two showing a significant increase with supplementation. Most of the studies reporting no effect of the supplementation on PTH, also noted no effect on calcium and phosphate concentrations. Only one study measured FGF23 and found no change. Bone turnover markers were only reported in two studies. Alkaline Phosphatase (ALP), C-terminal telopeptide (CTx) and Tartrate-resistant acid phosphatase 5b (TRAP5b) were shown not to change with supplementation. One study reported a significant improvement on the endothelium dependent microcirculatory vasodilation and other markers of vascular function.

### RCTs with Calcifediol Supplementation

In recent years, preparations of the 25 hydroxylated form of vitamin D, i.e. 25(OH)D or calcifediol were developed for oral administration. There are three forms; calcifediol and the extended release (ER) formula, provided as capsules and the immediate release (IR), provided as a liquid or capsule. The pharmacokinetic profile differs from the parent compound vitamin D. Intestinal absorption of 25(OH)D is known to be more efficient and is not dependent on fat absorption, the increase in plasma 25(OH)D is more rapid and the dose–response higher than that of the parent compound. The IR calcifediol formulation was approved in the USA in 1980 for treatment of CKD–MBD in dialysis patients [47]. However, it was withdrawn from the market in 2002, since it failed to show meaningful reduction of PTH (≥ 30%) in patients with CKD G3–4. IR calcifediol is still available in Europe and licensed for use in various conditions including vitamin D deficiency rickets, renal osteopathy and hypocalcaemia [47]. ER formulations of 25(OH)D are only available in the USA at the moment [47]. In 2016 ER calcifediol was approved in the USA to treat SHPT in adult CKD patients G3–4 and vitamin D insufficiency [47]. Studies in CKD patients showed that ER calcifediol results in a slower increase of 25(OH)D levels, more significant suppression of iPTH and less of an increase of 24,25(OH)D compared to IR-calcifediol [48].

Three ER calcifediol and one calcifediol study in CKD patients (G2–4) published since 2003 were found in our systematic search [22, 48–50] (Table 1). Dosages and durations of treatment varied but a significant dose-dependent increase in 25(OH)D and a decrease in iPTH was seen after oral administration in all studies [22, 48, 49]. A meta-analysis was not conducted due to limited numbers of studies and reported data.

ER calcifediol showed a significant but gradual increase of serum 25(OH)D in all three of the studies in contrast to the sharp increase with intra-venous administration of calcifediol. Intra-venous administration did not significantly suppress iPTH but there was an increase in 24,25(OH)_2_D (24,25 dihydroxy vitamin D), a catabolic product of 25(OH)D. ER calcifediol administration was associated with unaltered plasma calcium and phosphate concentrations in all studies and FGF23 concentrations in the 2 studies that included this measurement.

### RCTs: with Calcitriol and Vitamin D Analogues

The active form of vitamin D and its analogues used in the treatment of CKD patients include calcitriol and the vitamin D analogues paricalcitol (19-nor-1,25(OH)_2_D_2_) and the 1,25(OH)_2_D precursor alfacalcidol (1a(OH)D_3_) [51]. 1,25(OH)_2_D_3_ is identical to the endogenous activated form of calcifediol (25(OH)D_3_). Paricalcitol and alfacalcidol, the latter of which requires hepatic hydroxylation at the 25 position, are synthetic analogues of vitamin D [51] and are also referred to as vitamin D receptor activators (VDRA). VDRAs have been used for the management of SHPT in CKD patients for a few decades [52] and show to have reno-protective properties such as reducing albuminuria, renal damage and dysfunction [27, 28, 53]. Paricalcitol suppresses PTH secretion whilst it has a lower stimulatory effect on intestinal absorption of calcium and phosphate compared to 1,25(OH)_2_D [28]. Paricalcitol is also associated with reduction of cardiovascular events [30], although sufficient studies with CKD patients are still lacking.

All twelve studies included CKD patients G3–4, however, there were differences between studies in patient characteristics. For example, diabetes, established SHPT, use of phosphate binders and proteinuria. The duration of administration varied from 2 to 48 weeks and the dosages used in these studies from 0.25 to 2 μg.

All twelve RCTs showed a significant reduction in plasma PTH after administration of paricalcitol or 1,25(OH)_2_D [19, 26, 28, 30, 43, 46, 50, 53–58] (Table 1). This was also seen in a study that combined 1,25(OH)_2_D and Vitamin D_2_ [21]. Meta-analysis of the studies for which sufficient detail was available, confirmed this finding but heterogeneity was high (Fig. 2b). The effect of paricalcitol on serum 25(OH)D and 1,25(OH)_2_D concentrations were inconsistent. Two of the studies with paricalcitol [26, 55] reported a reduction of 1,25(OH)_2_D after supplementation.

Several studies compared different forms of supplementation. Kovesdy et al. compared the effects of vitamin D_2_ compared to paricalcitol in vitamin D deficient CKD patient with SHPT [46]. In both treatment groups, 25(OH)D significantly increased, but only in the paricalcitol group there was a significant decrease in PTH. Coyne’s study compared the effects of 1,25(OH)_2_D with paricalcitol [58]. In both treatment group there was a significant decrease of PTH. However, paricalcitol appeared to be suppress PTH more compared to 1,25(OH)_2_D (− 52% and − 46% PTH reduction, respectively). Susantitaphong et al. examined the effect of Vitamin D_2_ with or without 1,25(OH)_2_D and found a suppression of PTH only in the combined group [21]. Levin et al. [50] compared the effects of oral calcifediol with 1,25(OH)_2_D. Both treatment groups had a significant reduction of PTH, which was larger in the calcifediol group. A significant increase in 25(OH)D was shown in the calcifediol group, whilst there were no differences between the groups in the 1,25(OH)_2_D concentration. Meta-analysis comparing the effects of calcitriol or vitamin D analogues versus the administration of the precursor calcidiol or vitamin D on PTH, showed no significant difference between these forms. However, this analysis was limited to 4 studies for which sufficient detail was available and heterogeneity was high (Fig. 2c).

Inconsistent results were reported for serum/plasma calcium (four increase; six unchanged) and phosphate (two increase; eight unchanged) concentrations after calcitriol of analogue administration. Most of the studies reported no cases of hypercalcemia. Only one study reported significant higher incidence of hypercalcemia in the intervention group compared to placebo (22.6% and 0.9%, respectively) [30]. An increase in FGF23 after vitamin D analogues (paricalcitol) administration was found in all three studies reporting this outcome and in two of these studies, also a decrease in renal function was reported [26, 28, 55]. An increase in FGF23 was not reported in the four studies with Vitamin D or calcifediol and one with 1,25(OH)_2_D. Meta-analysis of the FGF23 response to vitamin D or analogue administration was not conducted due to the low number of studies (3) and lack of sufficient information provided (e.g. not all manuscripts reported whether intact or c-terminal or intact FGF23 was measured).

All four studies that measured either alkaline phosphatase (ALP) or bone specific (BALP) after paricalcitol treatment reported a decrease in this marker of bone metabolism.

Further outcomes included markers of endothelial and cardiovascular function, insulin sensitivity and proteinuria and were inconsistent between studies.

## Discussion of Clinical Trials of Vitamin D in CKD Patients

As expected, 25(OH)D increased after supplementation, but the vitamin D-25(OH)D dose–response appeared to be lower than in healthy people. Individual participant data (IPD)-level meta-analysis to characterize this relationship in CKD patients would be helpful to underpin the evidence base of vitamin D requirements to prevent and correct vitamin D deficiency in CKD patients.

The effect of vitamin D on PTH concentrations was inconsistent between individual studies and meta-analysis showed a non-significant suppressive effect. Comparing these studies with a meta-analysis of the PTH response in studies with participants not selected on the basis of their renal function [59, 60], results in CKD patients are less consistent. In these meta-analyses which included predominantly participants without renal impairement, a suppression of PTH was found in the majority of studies and meta-analyses found an overall reduction in PTH after vitamin D supplementation with generally lower dosages of vitamin D supplementation compared to dosages used in CKD patients. To address this, IPD-level meta-analysis of existing data and a vitamin D dose-ranging study in CKD patients with and without SHPT is urgently required to characterize the response of PTH to vitamin D treatment. This research would also facilitate the identification of patient groups that do and do not respond with a decrease in PTH to supplementation.

Administration of calcifediol was reported in four studies and all reported suppression of PTH. This may seem surprising since it requires renal activation to 1,25(OH)_2_D. Although it is difficult to compare these results to the administration of vitamin D, the use of calcifediol to suppress PTH in CKD patients holds promise since the risk of over suppression of PTH and hypercalcaemia appears less likely compared to calcitriol and paricalcitol.

We found that treatment with calcitriol and paricalcitol was associated a consistent and greater suppression of PTH. Increased risk of hypercalcaemia was only found in 1 of the 12 recent studies included in this review. IPD-level meta-analysis comparing calcifediol with vitamin D and calcifediol with calcitriol and its analogues may provide evidence of their relative effects. This may aid the incorporation of calcifediol in guidelines and provide alternative options for patients that might be prescribed calcitriol and paricalcitol.

An increase in FGF23 after treatment with vitamin D analogues was observed in all 3 studies reporting this outcome, but was unaltered in 4 studies with Vitamin D or calcifediol. This warrants further attention. FGF23 is a phosphaturic hormone, reducing phosphate reabsorption from the glomerular filtrate through downregulating the available sodium phosphate cotransporters [61]. FGF23 also downregulates the expression of 1a(OH)ase, suppressing the production of renal 1,25(OH)_2_D and upregulates 24(OH)lase increasing 1,25(OH)_2_D catabolism [61]. FGF23 is concentration-dependent and positively correlated with CKD progression, heart failure, vascular calcification, left ventricular hypertrophy and mortality in CKD patients [62]. These effects are partly thought to be caused by increased calcium retention caused by increased FGF23 and PTH concentrations [62], together with elevated aldosterone concentrations found in CKD patients due to activation of the Renin–Angiotensin–Aldosterone System (RAAS) [63]. High circulating aldosterone may enhance the effect of FGF23 on sodium retention in CKD patients and sodium and volume retention further contributes to the risk of vascular calcification [62]. An increase in both intact and c-terminal FGF23 with vitamin D supplementation is also reported in a meta-analyses of trials with deficient and healthy individuals [64], but so far not linked to increased risk of CVD events [64]. It was also earlier reported in CKD patients [39]. An increase in FGF23 may be an undesirable side effect of administration of vitamin D and its analogues and a better understanding of its effects with different forms of vitamin D is required, especially in CKD patients, in relation to their already increased FGF23 concentrations, alterations in vitamin D metabolism and increased risk of CVD.

Few RCTs reported the effect of vitamin D on markers of bone metabolism and variations in the range of markers prevented direct comparisons. However, all four studies that measured either ALP or BALP after paricalcitol treatment reported a decrease in this marker indicative of increased bone turnover in CKD patients.

Several factors may have influenced the overall findings. These include form, frequency and dosages of vitamin D and its analogues used. The selection of patient population, co-morbidities and their use of other medication will have influenced the response to supplementation. Further, the method used for estimating renal function may have influence the selection of study participants.

### Methods for Estimating Renal Function

All studies included in this review used eGFR as a measure to assess kidney function. None applied a direct method. Most studies used creatinine based algorithms, and the majority the Modification of Diet in Renal Disease 4-variable (MDRD-4) equation, except Zoccali et al. [55] who used the Chronic Kidney Disease Epidemiology Collaboration (CKD–EPI) Creatinine-Cystatin C algorithm, that incorporates both creatinine and cystatin C. Plasma creatinine is significantly affected by age, nutrition, gender, physical activity and muscle mass [65, 66]. Also, Agarwal et al. showed that short-term paricalcitol treatment in CKD patients can increase serum creatinine and creatinine excretion without altering creatinine clearance [65, 66]. MDRD-4 is the most commonly used formula for eGFR in medical practice. However, there are differences between guidelines. The UK National Institute of Health and Care Excellence (NICE) recommends the use of CKD–EPI for the majority of patients [67], but for the assessment of eGFR in those with CKD stage 3a (eGFR 45–59 mL/min/1.73 m^2^) and no proteinuria, the use of cystatin C-based equations is recommended [68]. Also the Kidney Disease Improving Global Outcomes (KDIGO), National Kidney foundation (NKF) and Caring for Australasians with Renal Impairment (CARI) guidelines recommend the use of CKD–EPI. The Cockroft–Gault algorithm may be the preferred option for the older population since this incorporates body size [69].

Cystatin C is a relatively new biomarker. Cystatin C based eGFR has been reported to correlate better with mortality risk factors in CKD patients than creatinine based eGFR [65, 66]. Cystatin C is filtered by the glomerulus, is not secreted by the renal tubules and it is generated at a constant rate by all cells in the body [65, 66]. There are two formulae based on cystatin C; the CKD–EPI using cystatin C and CKD–EPI using a combination of cystatin C and creatinine [65, 66].

There are considerable differences in the resulting eGFR value and CKD classification [69] and accordingly, the choice of the method may have influenced the characteristics of patients included in studies.

### Medication Use and Vitamin D

Many drugs used by CKD patients to manage symptoms of CKD and CVD influence vitamin D metabolism and synthesis, although the mechanisms are still largely unknown [70]. The most commonly used therapies for CKD patients involve angiotensin inhibitors (ACE), aldosterone receptor antagonists (ARAs) and receptor blockers [70]. They are used to inhibit the RAAS [71]. Statins are also frequently used [72]. There are conflicting results on the association between these medications and vitamin D status. Yuste et al. found significant lower 25(OH)D levels in patients treated with statins compared to the patients treated with ACE inhibitors or ARAs [70]. In the same study they found higher 25(OH)D levels in patient treated with xanthine oxidase inhibitors (medication for hyperuricemia) [70]. On the other hand, a different study showed no significant associations between concentrations of 25(OH)D and treatment with statins, ACE inhibitors and/or ARAs [73]. The use of these medications is however seldomly reported in RCTs.

Medication use or dietary strategies to manage hyperphosphatemia in CKD can also have influenced the findings and the reported side effects in these studies. Hyperphosphatemia is usually managed by dietary restriction of phosphate intake or prescription of phosphate binders [74] to decrease the availability of phosphate for intestinal absorption. There are three types of phosphate binders available: containing calcium, aluminum or non-calcium containing binders. Calcium based binders can be used as the initial binder therapy in CKD patients but are not the preferred option in case of hypercalcemia and/or when plasma PTH concentrations are < 150 pg/mL on two consecutive blood tests [75]. Calcimimetics are allosteric activators of the calcium sensing receptor of parathyroidal cells [51]. Calcimimetics are usually prescribed in CKD patients with SHPT to activate calcium receptors and thus suppressing PTH [51] and are used when vitamin D analogues have failed to reduce SHPT. Calcimimetics in combination with active vitamin D therapy are used to reduce the risk of developing hypercalcemia and hyperphosphatemia [76].

A secondary analysis of a large study showed that the choice of phosphate binders may significantly impact vitamin D metabolism and may influence the safety profile of vitamin D administration. In the Phosphate Normalization Trial on CKD patients [93], participants were randomized to receive either sevelamer carbonate, lanthanum carbonate, calcium acetate for 9 months after which vitamin D metabolites were measured. In the group taking calcium acetate, 24,25(OH)_2_D, the vitamin D metabolite ratio (VMR) [24,25(OH)_2_D:25(OH)D] increased and 1,25(OH)_2_D decreased [77]. Also, the group taking sevelamer carbonate had an increased 24,25(OH)_2_D and VMR, but this was lower than in the calcium acetate group [77]. No changes in 1,25(OH)_2_D were reported [77]. In the group taking lanthanum there we no changes in vitamin D metabolite concentrations.

In the studies included in this systematic review, patients were included that did or did not use phosphate binders and/or calcimimetics. No clear pattern in the response or incidence of hypercalcemia appeared amongst these studies.

## Summary of Published Guidelines

### Guidelines for Dietary Vitamin D Intakes and Supplementation for Population Health and Patient Management

#### General Population Requirements and Recommendations

Population guidelines for dietary vitamin D intakes are partly based on the required intakes to prevent vitamin D deficiency or to achieve pre-defined target ranges of 25(OH)D or sufficiency. Also evidence from RCTs and other research designs linking vitamin D intake and status to health outcomes are considered. Thresholds for deficiency, i.e. the plasma concentration of 25(OH)D below which the risk of disease increases, are predominantly defined on the basis of skeletal health outcomes, but in some guidelines also other health outcomes are considered. In most guidelines, minimal contribution of vitamin D synthesis in the skin is assumed. There is considerable variation in the definition and thresholds or ranges of 25(OH)D concentration that encompasses vitamin D sufficiency or target ranges of 25(OH)D between health authorities. In addition, strategies (e.g. type of data considered) to set requirements differ between health authorities. Review of approaches are beyond the scope of this review and are provided in Bouillon 2017 [78].

Dietary reference values (DRV) or equivalents, defined by different public health institutes vary as a consequence of differences in the defined threshold for deficiency or target values for 25(OH)D and by age group or physiological state and are summarized in Table 3 [8, 79, 80]. The umbrella term DRV is used in Europe and World Health Organization/Food and Agriculture Organization (WHO/FAO) and similarly, dietary reference intake (DRI) in the US. These comprise a range of nutrient values that apply to the general population or specific population groups [8, 79–81]. The DRV includes the Reference Nutrient Intake (RNI) which is the intake to meet the requirements of 97.5% of the population. There where this cannot be established, an Adequate Intake (AI) is defined. Similarly in the US, the recommended dietary allowance (RDA) represents the requirements of 97.5% of the population [82]. Population guidelines differ from clinical guidelines as the latter consider altered dietary requirements associated with underlying conditions.

**Table 3:** Population daily Reference Nutrient Intake (RNI) or Recommended Dietary Allowance (RDA) or equivalents for vitamin D according to different countries and organizations Ψ

Country/organization	Adults IU/d (μg/day)	Older than 65 years IU/d (μg/day)	Deficiency of 25(OH)D [78]
Nordic countries [95]	400 (10)	400-800 (10–20)	25–30 nmol/L
UK [96]	400 (10)	400 (10)	25 nmol/L
Ireland [97]	0-400 (0–10)	400 (10)	30 nmol/L
Netherlands [98]	0-400 (0–10)	800 (20)	25–30 nmol/L
Belgium [98]	400-600 (10–15)	600 (15)	
France [98]	200 (5)	400-600 (10–15)	
DACH [98]	800 (20)	800 (20)	25–30 nmol/L
Spain [98]	600 (15)	600 (15)	
Australia and New Zealand [86]	600 (15)	600-800 (15–20)	25–30 nmol/L
EFSA 2017 [81]^*^	600 (15)	600 (15)	50 nmol/L*
Institute of Medicine [82]	600 (15)	800 (20)	30 nmol/L
WHO/FAO	200 (5)	200 (5)	25 nmol/L

After Lips et al. [79] and Bouillon [78]

*Adequate intake set for vitamin D intake based on target value of 25(OH)D; Ψ to convert from IU to μg divide by 40

Although vitamin D deficiency is one of the most common nutritional deficiencies in the world, no routine screening for the general population and most patient groups is recommended [9, 67, 81, 82]. Only people at high risk or with clinical features of vitamin D deficiency are recommended to be tested [83]. For example, KDIGO working group and NKF recommend annual screening for vitamin D deficiency for CKD patients [84]. CKD patients are classified as a high risk group due to their dietary modification (restricted protein intake), advice to restrict sunlight exposure and reduced cutaneous synthesis associated with common CKD comorbidities and renal losses.

#### Guidance for Patient Management and CKD Patients

Clinical guidelines for patient management or for specific patient groups provide guidance for the prevention and correction of vitamin D deficiency. These are partly based on population guidance or define specific target values for 25(OH)D and vitamin D intakes, based on altered supply or bio-availability, metabolism and/or requirements. The UK NICE [67] and the US Endocrine Society [9] offer guidelines for prevention and treatment of vitamin D deficiency in patients, including CKD patients. The UK Royal Osteoporosis Society (ROS) [85] provides guidelines that focus on patients with osteoporosis and for osteomalacia and osteoporosis prevention (Table 4). Specific guidance for the management of CKD–MBD were developed by the NKF, the KDIGO CKD–MBD Guideline [84] and CARI [86–88]. Approaches and criteria used are discussed in more detail below.

**Table 4:** Guidelines for the correction of vitamin D deficiency for patient management (general and for specific groups)

Endocrine society [9]	NICE [67]	ROS [85]
*Dosage schemes for the correction of vitamin D deficiency*		
Sufficient: > 75 nmol/L Insufficient: 50–75 nmol/L Deficiency: < 50 nmol/L	Sufficient: > 50 nmol/L Insufficient: 25–50 nmol/L Deficiency: < 25 nmol/L	Sufficient: > 50 nmol/L Insufficient: 25–50 nmol/L Deficiency: < 25 nmol/L
Dietary intake for patients at risk: 19–70 year 600 IU/day; > 70y 800 IU/day Treating vitamin D deficiency in adults: 50,000 IU/week for 8 weeks or 6000 IU/day Followed by maintenance therapy of 1500–2000 IU/day	Vitamin D_3_ is the preferred form of supplementation to treat vitamin D deficiency Vitamin D deficiency treatment: Fixed loading dose of vitamin D up to total of 300,000 IU, split dose either weekly or daily Followed by lifelong maintenance treatment of 800 IU/day	Vitamin D_3_ is recommended for treating vitamin D deficiency Vitamin D deficiency treatment: fixed loading up to a total of 300,000 IU given either as weekly or daily split doses Maintenance therapy: started one month after loading with doses equivalent to 800–2000 IU/day (maximum 4000 IU/day) given either daily or intermittently

No specific guidelines for CKD patients are included. Endocrine society and ROS are led by clinical experts and NICE is a government led committee

**Fig. 3:**
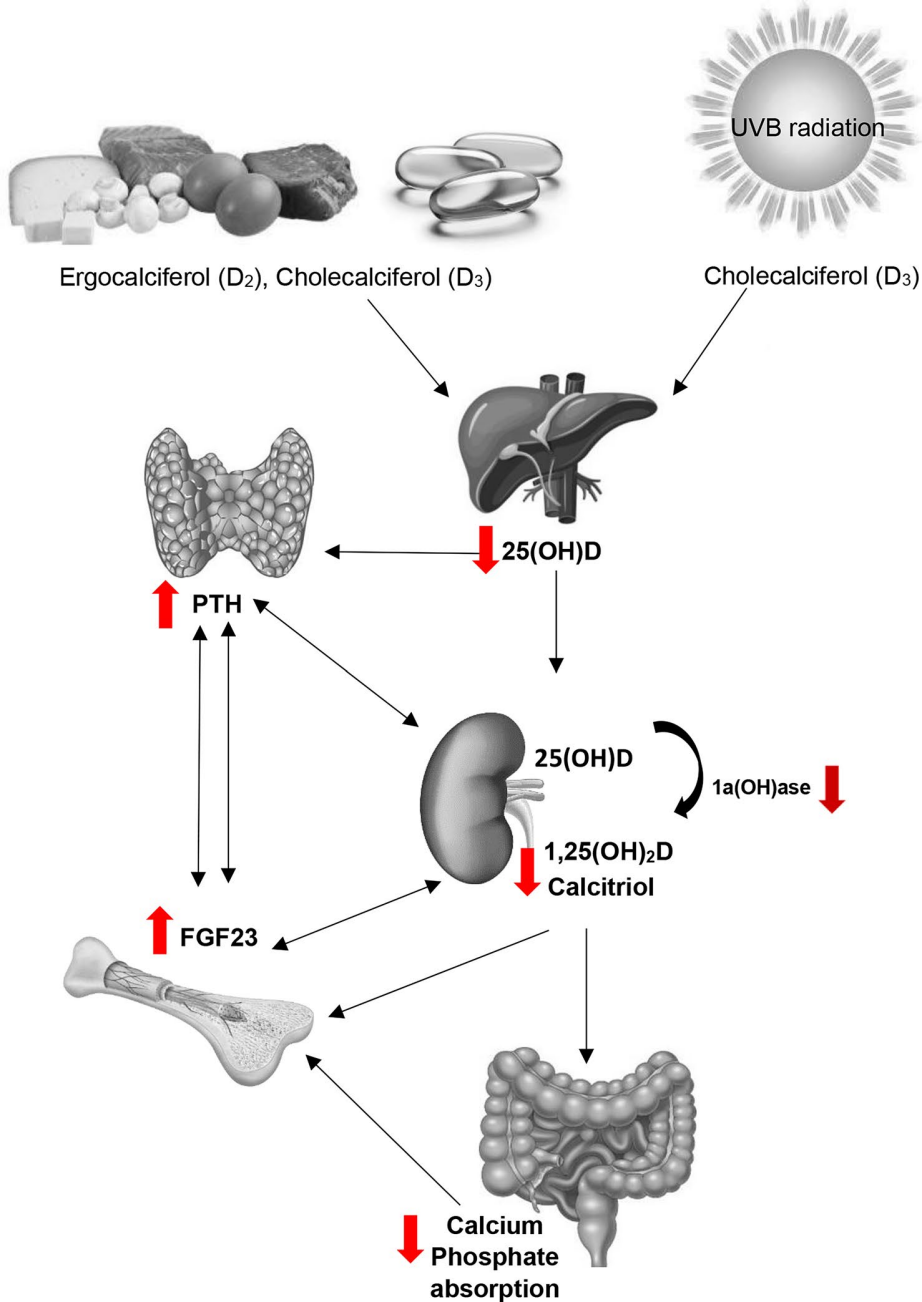
Changes in Vitamin D metabolism and the renal-bone axis with CKD. Arrows indicate direction of changes with CKD. With chronic kidney disease the combination of limited vitamin D intake and reduced renal capacity to activate 25(OH)D to 1,25(OH)_2_D results to a chain reaction of changes in metabolism. A decrease in 1,25(OH)_2_D results in a decrease in intestinal absorption of calcium. This stimulates PTH secretion which in turn increases bone resorption. Phosphate retention due to reduced kidney filtration capacity further stimulates PTH and the production and release of FGF23 from bone cells to increase renal phosphate excretion

The NKF and KDIGO guidelines cross-refer to guidelines for the general population, especially for patients in early stages of CKD. Specific guidelines for patients with advanced renal impairment include the use of vitamin D analogues [84]. These guidelines, including the daily recommended vitamin D intakes are detailed in Tables 5 and 6 by CKD category. Additional recommendations are in place for patients with CKD 3–4. For these patients, it is recommended that with vitamin D supplementation, plasma calcium and phosphate should be monitored and supplementation dose should be adjusted when required [84] (Fig. 3).

**Table 5:** Guidelines for monitoring and correction of vitamin D deficiency in CKD patients

	G1	G2	G3	G4	G5
	≥ 90 mL/min	60–89 mL/min	30–59 mL/min	15–29 mL/min	< 15 mL/min
NKF KDOQI (2003) [75] (2016) [84]	Follow guidelines for general population Target thresholds as general population		If plasma/serum 25(OH)D concentration is < 75 nmol/L D_2_ supplementation should be given with dosages dependent on baseline values (Table 4) with monitoring of 25(OH)D and calcium and phosphate homeostasis^†^ (Fig. 1) Maintenance: continue supplementation with a vitamin-D containing multi-vitamin and an annual reassessment of 25(OH)D^†^ ER calcifediol can be used with vitamin D deficiency and SHPT		Vitamin D analogue therapy should be given when SHPT is progressive and persistent higher from the upper limit of the assay used
KDIGO (2009) [1] (2017) [90]	Follow guidelines for general population Target thresholds for 25(OH)D as for general population Monitor plasma/serum 25(OH)D concentrations once a year. If normal no treatment is required. If deficient, treat per general population^‡^		Monitoring of plasma/serum 25(OH)D concentrations at intervals dependent on CKD stage, baseline values and therapeutic interventions*, with monitoring of calcium and phosphate homeostasis* Vitamin D deficiency and insufficiency be corrected using treatment strategies recommended for the general population In non-dialysis patients with progressively rising PTH concentrations above the upper limit of normal for the assay, despite correction of modifiable factors the use 1,25(OH)_2_D or vitamin D analogues is recommended With severe and progressive SHPT and CKD-MBD in G4-5 1,25(OH)_2_D or vitamin D analogues is recommended		
Kidney Health Australia (CARI 2012–2013) [86, 87]	Vitamin D deficiency (< 37.5 nmol/L) and insufficiency (37.5–75 nmol/L) should be corrected using treatment strategies recommended for the general population Vitamin D therapy for early CKD patients with SHPT is recommended with monitoring of markers of calcium and phosphate homeostasis and bone metabolism^§^ 25(OH)D and PTH levels should be monitored regularly whilst on vitamin D therapy^§^				

^†^Monitor phosphorus and corrected total calcium every 3 months. See Fig. 1 for more details [75, 84]

^‡^Monitor calcium and phosphate levels. Provide instructions to reduced dietary phosphate intake [1, 90]

*Monitoring intervals: G3: serum calcium and phosphorus: every 6–12 months; intervals for PTH based on baseline concentration and CKD progression; G4: serum calcium and phosphorus: every 3–6 months; PTH: every 6–12 months; G5 to 5D: serum calcium and phosphorus: every 1–3 months; PTH: every 3–6 months [1, 90]; ^§^CKD patients on vitamin D therapy: regular monitoring of plasma calcium, phosphate and alkaline phosphatase levels [86, 87]. For abbreviations, see Table 1

**Table 6:** Guidelines correction and monitoring of vitamin D deficiency in patients with CKD 3 and 4

Serum 25(OH)D nmol/L [ng/mL]	Definition	Vitamin D_2_ dose	Duration (months)	Comment
< 12 [< 5]	Severe vitamin D deficiency	50,000 IU/w orally × 12 weeks; then monthly	6 months	Measure 25(OH)D levels after 6 months
		500,000 IU as a single I.M. dose	n/a	Assure patient adherence; measure 25(OH)D at 6 months
12–37 [5–15]	Mild vitamin D deficiency	50,000 IU/w × 4 weeks; then 50 000 IU/m orally	6 months	Measure 25(OH)D levels after 6 months
40–75 [16–29]	Vitamin D insufficiency	50,000 IU/m orally	6 months	n/a

After NKF KDOQI (2003): Guideline 7;Table 26 [75]

The US NKF developed the Kidney Disease Outcomes Quality Initiative (KDOQI), a specific CKD–MBD guideline (Clinical Practice Guidelines for Bone Metabolism and Disease in Chronic Kidney Disease), published in 2003 [75]. This guideline includes recommendations for the management of vitamin D deficiency and SHPT, calcium and phosphate metabolism. Also the effects of vitamin D deficiency and supplementation on bone metabolism and disease were considered (Table 5). The guideline was predominantly based on evidence on the prevention and management of vitamin D deficiency and the progressive increase of PTH. It is acknowledged that there is a lack of high quality evidence for bone health or patient orientated clinical outcomes, such as mortality and cardiovascular disease risk. Part of this guidance was revised by NKF in 2016 [84] focusing on vitamin D deficiency and SHPT in CKD stages 3–4. In this updated guidance, targets for intact PTH thresholds by CKD category, as defined in the 2003 guideline were removed [84], due to lack of evidence of benefit. Specific dosage schemes for the prevention of vitamin D deficiency for CKD patients are not given in the NKF guidelines 2003 [75] (Table 5), but for the correction of deficiency, recommendations on dosages and duration are provided (Table 6). Vitamin D analogues are recommended only for patients with a progressive increase in PTH and for the treatment of SHPT. The 2016 update highlighted the potential benefits of the use of oral ER 25(OH)D in CKD patients. Few studies were available at that time to support this. In our systematic review, additional studies using this form of vitamin D are included. In 2017, a commentary was published, reflecting the views of the KDOQI CKD–MBD work group which were mostly in agreement with the updated KDIGO guidelines discussed below [89].

In 2003, the first detailed guidelines by KDIGO was published [1]. In 2017 KDIGO issued their updated guideline for CKD–MBD [90] but little detail on the response to vitamin D supplementation on PTH and bone turnover markers was included. In parallel to the NKF guideline, there are no specific recommendations (type of vitamin D, dose and duration) for the prevention of vitamin D deficiency and the treatment of SHPT due to a lack of sufficient high quality evidence specific to CKD patients. This guideline emphasizes the importance of managing other factors influencing PTH, including a high plasma phosphate and low calcium. This recommendation also considered reported adverse effects (since the previous review) of vitamin D analogues and calcitriol on the development of hypercalcemia, whilst clinically relevant outcomes did not substantially improve. It was therefore concluded that the risk–benefit ratio of treating an elevated PTH with these forms of vitamin D is no longer favourable for the majority of patients [75]. Therefore, in this guideline, the use of vitamin D analogues and 1,25(OH)_2_D are recommended only for patients with severe and progressive SHPT (Table 5).

The Kidney Health Australia, CARI guidelines of 2012–2013 [86, 87] and the CKD guidelines for general practice issued in 2015 [88] recommend that vitamin D deficiency in CKD patients should be corrected following guidelines for the general population (Tables 3, 5). Vitamin D therapy on prescription is recommended for early stages of CKD with SHPT, with regular monitoring of plasma calcium, phosphate, PTH, alkaline phosphatase and 25(OH)D. Treatment with 1,25(OH)_2_D is only recommended in later stages of CKD for the treatment of SHPT.

#### Thresholds and Correction of Vitamin D Deficiency

Thresholds for the definition of vitamin D deficiency for the general population differ between advisory bodies (summarized in Table 3). Some, but not all, also provide thresholds of sufficiency. Recommended 25(OH)D target concentrations and thresholds for deficiency for specific patient groups may be higher than for generally healthy people (Tables 4, 6). Guidance for the correction of vitamin D deficiency is not provided in population guidance, but instead relies on country-specific clinical guidance for patient management. Tables 4 and 5 provides an overview of the recommendations from 4 authorities, including the NKF [9, 86].

There is considerable between-person variation in the dose–response to vitamin D because of the numerous factors that can affect plasma 25(OH)D and its increment after intake. Reported increases in plasma 25(OH)D in apparently healthy populations (including those that were deficient at baseline) range from 1.1 to 5.75 nmol/L per 100 IU/day [91–94]. This dose–response relationship is influenced by baseline concentrations of 25(OH)D, vitamin D dose, frequency of administration, body composition and a number of medical conditions [47]. Population guidance for the prevention of deficiency allows for these variations, but specific at risk groups may need higher intakes to prevent and correct vitamin D deficiency. For the correction of deficiency, higher intakes are required and often loading dosages are recommended, followed by maintenance therapy (Table 4). Clinical monitoring is required with these loading dosages.

Population guidance considered at what concentration range of 25(OH)D, the risk of SHPT was increased and this is incorporated in the assessment of vitamin D requirements [8, 82]. Also this relationship is characterized by a large variability and target values for PTH were not formulated for the general population.

In CKD patients, the threshold for vitamin D sufficiency and the vitamin D intake to achieve and maintain sufficiency is less well established than in generally healthy people. This partly due to the fact that few high quality studies relating 25(OH)D with clinical outcomes, such as fragility fractures or bone mineral density, were conducted in CKD patients. Also, the heterogeneity in this patient group plays a role. In these patients, the vitamin D dose–response and relationship between 25(OH)D and PTH is dependent of CKD category, renal capacity to produce 1,25(OH)_2_D and the degree of PTH resistance. Also, diversity in clinical presentation of CKD–MBD, influences the response in bone metabolism.

**Fig. 4:**
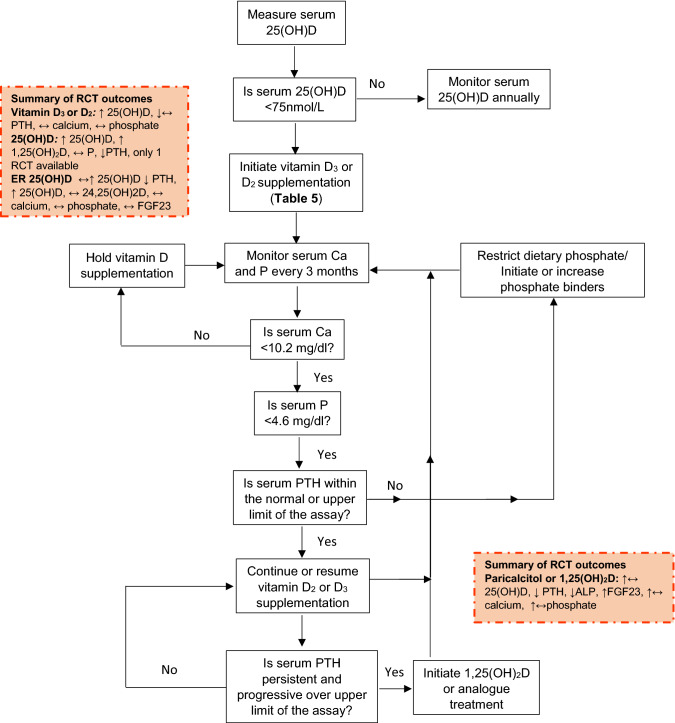
Guidance for monitoring of vitamin D status and supplementation and monitoring of calcium and phosphate metabolism in CKD stages G3–4. Based on recommendations from the different organizations summarized in Table 5. Adapted from: NKF KDOQI (2003) [75]. Finding in recent RCTs (2003–2020) included in the systematic review are summarized in yellow boxes. For abbreviations see Table 1

For CKD patients, a higher target concentration (> 75 nmol/L) than for the general population is recommended in the NKF and CARI guidelines [84, 86] with regular monitoring and correction as required on a 6–12 monthly basis (Tables 4, 5, 6 and Fig. 4). Target values for PTH could not be set on the basis of the evidence available. In addition, the PTH-25(OH)D relationship (used formulation of in many population guidelines) could not be used in the assessment of vitamin D requirements for CKD patients due to their altered relationship. Current recommendations advise regular monitoring and management if PTH progressively increases. In view of the variability in clinical presentation, patients are managed on a case-by-case basis with monitoring of calcium and phosphate homeostasis (Fig. 4).

Guidance for management of vitamin D status and deficiency, population recommendations and patient management are summarized in Tables 3, 4 and Fig. 4.

## Conclusion

Major gaps remain in the evidence base for the management of vitamin D status in relation to CKD–MBD, i.e. the dose–response, SHPT, altered bone metabolism, bone density and integrity and fracture risk. Recent studies included in this systematic review varied in design, vitamin D form used there was a high degree of heterogeneity with regard to duration, dose, and population characteristics. Our systematic review showed that the effect of vitamin D on PTH concentrations was inconsistent between studies and meta-analyses showed a non-significant reduction. This is in contrast to findings in studies in patients not selected on basis of pre-existing CKD. This is likely explained by the small sample size of studies and the fact that in some but not all patients with CKD, other drivers are predominant and prevent a decrease of PTH secretion. More consistent effects on PTH was found with calcifediol; all 4 studies that used this form reported a reduced PTH. Also treatment with calcitriol and paricalcitol was associated with a consistent suppression of PTH. An increase Fibroblast Growth Factor 23 (FGF23) after treatment with vitamin D analogues was observed in all 3 studies reporting this outcome, but was unaltered in 4 studies with Vitamin D or 25(OH)D. The increase in FGF23 with analogue administration warrants attention as this hormone is already elevated in CKD patients and is a predictor of vascular calcification and CVD. It’s increase may indicate an undesirable side effect of administration of these forms of vitamin D. Few RCTs reported the effect of vitamin D on markers of bone metabolism and variations in the range of markers prevented direct comparisons. However, all 4 studies that measured either ALP or BALP after paricalcitol treatment reported a decrease in this marker that is indicative of increased bone turnover in CKD patients.

Guidelines for the first stages (G1–G3a) follow general population recommendations for the prevention of vitamin D deficiency. For the correction of deficiency, general or CKD specific patient guidelines provide recommendations. These are summarized in a tabulated format to facilitate their use in clinical practice.

## Data Availability

All data are publicly available.
